# Chronic Restraint Stress Inhibits Hair Growth via Substance P Mediated by Reactive Oxygen Species in Mice

**DOI:** 10.1371/journal.pone.0061574

**Published:** 2013-04-26

**Authors:** Nan Liu, Lin-Hui Wang, Ling-Ling Guo, Guo-Qing Wang, Xi-Ping Zhou, Yan Jiang, Jing Shang, Koji Murao, Jing-Wei Chen, Wen-Qing Fu, Guo-Xing Zhang

**Affiliations:** 1 Department of Clinical Psychology, Medical College of Soochow University, Suzhou, P.R. China; 2 Department of Physiology, Medical College of Soochow University, Suzhou, P.R. China; 3 Department of Pathology, Medical College of Soochow University, Suzhou, P.R. China; 4 New Drug Screening Center, China Pharmaceutical University, Nanjing, P.R. China; 5 Department of Clinical Laboratory, Faculty of Medicine, Kagawa University, Kita-gun, Kagawa, Japan; 6 Department of Internal Medicine, Suzhou Chinese Traditional Medicine Hospital, Suzhou, P.R. China; University Hospital Hamburg-Eppendorf, Germany

## Abstract

**Backgrounds:**

Solid evidence has demonstrated that psychoemotional stress induced alteration of hair cycle through neuropeptide substance P (SP) mediated immune response, the role of reactive oxygen species (ROS) in brain-skin-axis regulation system remains unknown.

**Objectives:**

The present study aims to investigate possible mechanisms of ROS in regulation of SP-mast cell signal pathway in chronic restraint stress (CRS, a model of chronic psychoemotional stress) which induced abnormal of hair cycle.

**Methods and Results:**

Our results have demonstrated that CRS actually altered hair cycle by inhibiting hair follicle growth in vivo, prolonging the telogen stage and delaying subsequent anagen and catagen stage. Up-regulation of SP protein expression in cutaneous peripheral nerve fibers and activation of mast cell were observed accompanied with increase of lipid peroxidation levels and reduction of the activities of superoxide dismutase (SOD) and glutathione peroxidase (GSH-Px) in CRS mice skin. In addition, SP receptor antagonist (RP67580) reduced mast cell activations and lipid peroxidation levels as well as increased GSH-Px activity and normalized hair cycle. Furthermore, antioxidant Tempol (a free radical scavenger) also restored hair cycle, reduced SP protein expression and mast cell activation.

**Conclusions:**

Our study provides the first solid evidence for how ROS play a role in regulation of psychoemotional stress induced SP-Mast cell pathway which may provide a convincing rationale for antioxidant application in clinical treatment with psychological stress induced hair loss.

## Introduction

Hair is an important skin appendage whichhas wide range of functions including thermoregulation, physical protection, sensory activity, and social interactions [1]. Up to date, basic and clinical observations have demonstrated that psychoemotional stress was a potential cause of hair loss [2]–[5]. Further study revealed that hair loss was highly related with hair follicle pathophysiological changes [6]. Hair follicle has its own specific cycle which is defined as rapid growth (anagen), regression (catagen), and resting periods (telogen) according to histological morphology [7]–[10]. Numerous clues have revealed that psychoemotional stress played an important role in abnormal hair cycling [4]–[5], [11]–[12]; however, the mechanisms of psychoemotional stress induced alterations of hair cycling still remains many unknown elements.

Substance P (SP) is a stress-related neuropeptide, synthesized in dorsal root ganglion small neuronal cell body, and released from cutaneous peripheral nerve terminals [13]–[14]. As a member of neuropeptides family, there are three principal neurokinin receptors have been described: NK-1, NK-2, and NK-3, which bind with high affinity to SP. Remarkably, the NK1 receptor is widely distributed to the central and peripheral nervous system especially in mast cell in mammals [4], [15]–[18]. Previous studies have demonstrated SP induced neurogenic inflammation and mast cell degranulation which were involved in skin disorders, such as atopic dermatitis and psoriasis [4]–[5], [19]–[21]. Therefore, mast cell has been regarded as a “switchboards” of neurogenic inflammation induced by stress [14]. Arck and her colleagues proposed that SP was the key mediator of the “brain-hair follicle axis” through mast cell activation [4], [22]. Their further study clearly confirmed that the center position of substance P in regulating human hair follicle [23]. These observations indicated that the interaction between SP and mast cell via NK-1 receptor might be one of the most important pathways in stress which induced abnormal of hair cycle.

In recent decades, oxidative stress and excessive reactive oxygen species (ROS) generation, have been generally accepted, played a critical role in various diseases, including skin diseases [24]–[25]. Some allergic and inflammatory skin diseases, for example, atopic dermatitis, urticaria and psoriasis were demonstrated to be closely related to oxidative stress [26]–[27]. Several researches also revealed that the mast cell function could be modulated by the balance of oxidative/antioxidative system which could induce sensitization of mast cell by increase of cytokine mRNA expression [28]–[29]. In addition, previous study also confirmed that oxidative stress involved in skin and hair aging [30]–[31]. These studies indicated that oxidative stress may play a vital role in mast cell activation and hair follicle pathological change [32]. Recently, it was reported that ROS were second messengers of neurokinin signaling in peripheral sensory neurons [33]. Although acute and chronic psychoemotional stress both induced oxidative stress in many organs such as brain, liver, kidney, heart and stomach of rats [34]–[39], and SP-mast cell pathway was demonstrated to play a key role in psychoemontional stress induced abnormal of hair cycle. However, the role of oxidative stress in chronic psychoemotional stress induced abnormal of hair cycle, especially its role in SP-mast cell pathway in skin has not been investigated.

For this reason, the present study aims to explore the possible role of oxidative stress in SP-mast cell pathway in chronic restraint stress mice (CRS, a chronic psychoemoational stress model). We find that CRS could: (1) inhibit weight gain and increase plasma cortisol content; (2) inhibit the hair growth observed by visual and microscopic method; (3) induce increase of oxidative stress marker, such as lipid peroxidation levels determined thiobarbituric acid reactive substances (TBARS), and alter the activities of antioxidant enzymes, such as superoxide dismutase (SOD) and glutathione peroxidase (GSH-Px) in skin; (4) increase the number of SP immunoreactive nerve fibers and activate mast cell by observing immunofluorescence and toluidine blue stain in skin. (5) CRS-induced pathological changes in skin could be normalized by SP receptor specific antagonist (RP67580). (6) CRS-induced SP-mast cell pathway could be suppressed by antioxidant (Tempol), which also normalizes hair growth.

## Materials and Methods

### Animals

Six to eight weeks old male C57BL/6 mice (Provided by center of experimental animal, Soochow University) were used because the dorsal hair follicles in such strain mice at this age were in telogen stage naturally [9]–[10]. All mice were acclimated for a week under the following conditions: room temperature was 22±1°C; humidity was 50±5% and with a 12-hour light: dark cycle (lights on at 6:00 a.m. and off at 18:00 p.m.). During this period, food and water were provided *ad libitum*. The present study was conformed to the *Guide for the Care and Use of Laboratory Animals* published by the US National Institutes of Health (NIH Publication No. 85–23, revised 1996). All procedures were approved by the Animal Care and Use Committee of the University of Szeged.

### Anagen Induction and CRS Application

Method of depilation to induced anagen of hair cycle was as described previously [4]. Briefly, wax/rosin mixture (1∶1 on weight) was applied to the dorsal skin (from neck to tail) of mice, then waiting for it dried and peeling-off the wax/rosin mixture removed all hair shafts and immediately induced a highly synchronized hair growth, as evidenced by the homogeneously pink skin color in the back, which indicated all hair follicles in telogen [8]–[10]. CRS application began on day 1 after depilation. Following the previously reported method, mice were placed in a 50 mL conical centrifuge tube with multiple punctures to allow ventilation, then plugged the tube to prevent shuttle of mice [40]. Tubes were held slantly (head elevation) in the cage. Application of CRS was carried out for 6 hours daily (10:00 a.m.–16:00 p.m.) while food and water were prohibited. Period of CRS was lasted for 9 and 18 days and the control mice were kept in their original cage, but food and water were not provided during the CRS period.

### Reagents

RP67580 (Santa Cruz biotechnology,CA, USA),a selective antagonist of the tachykinin NK1 receptor [41]–[42] was dissolved in ethanol and then diluted with PBS to make a final concentration of 1 mg/ml. RP67580 was injected intraperitoneally at a dose of 200 µg/mouse every other day [4]. 4-Hydroxy-2, 2, 6, 6-tetramethyl piperidinoxyl (Tempol, Sigma, MO, USA) is a stable membrane-permeable superoxide dismutase (SOD) mimetic that exhibits potent antioxidant activity against superoxide as well as hydroxyl radicals [43]. Our previous studies have demonstrated that Tempol decreased vascular superoxide anion production in conscious chronic and acute angiotensin II induced hypertensive rats [44]–[45]. In the present study, Tempol was injected intraperitoneally to against oxidative stress in skin at a dose of 200 mg/kg/day because that the maximum tolerated dose of Tempol administered intraperitoneally was found to be 275 mg/kg [46]. The mice were randomly divided into the following four groups: (1) Control group; (2) CRS group: no drug injection and application of CRS; (3) Tempol group: application of CRS concomitant with 200 mg/kg/day of Tempol injection intraperitoneally for 9 days or 18 days; (4) RP67580 group: application of CRS concomitant with 200 mg/mouse of Tempol injection intraperitoneally for 9 days or 18 days (every other day).

### Tissue and Plasma Preparation

In order to explore the change of whole hair cycle, we chose two time points which indicted different hair cycle stage. The first step, on day 9 after depilation when hair cycle of control mice was in the late anagen [9]–[10], blood samples were collected by enucleated eyeball under anesthesia and added heparin for anticoagulation, then centrifuged at 3000 rpm for 15 minutes to obtain plasma, after that stored at−80°C immediately. Skin specimens from back skin were harvested about 2×5 cm, the region of neck, which was an essential section for the quantitative histomorphology of the hair cycle [4]. It was fixed in 4% paraformaldehyde then paraffin-wax embedded. The remaining skin specimens were cryopreserved at−80°C immediately. The second step, on day 19 after depilation when hair cycle of control mice just spontaneously entered the catagen transformation [9]–[10], mice were sacrificed; plasma and skin tissues were sampled according to above mentioned method.

### Measurement of Body Weight and Plasma Corticosterone Concentration

All mice were recorded body weight on day 0, 4, 9, 14, 19 after depilation. Plasma corticosterone concentration was determined by mouse corticosterone RIA kit following manufacturer's instruction (Beyotime, China).

### Protein Concentration Measurement

The skin tissues were homogenized in a solution containing 0.15 mol/L KCl and 0.02 mol/L Tris-HCl (pH 7.4) then used BCA Protein Assay Kit (Beyotime, China) to determined protein concentration. Finally, the skin tissue homogenates were adjusted at a concentration of 1 mg/ml for further analysis.

### Measurement of Lipid Peroxidation Levels in Skin

Thiobarbituric acid reactive substances (TBARS) is a well-recognized parameter for lipid peroxidation levels which is also as a biomarker of tissue damage caused by oxidative stress [47]. The method for measurement of TBARS levels has been described previously [41]–[42]. Briefly, 100 µL sample solution was mixed with 15% trichloroacetic acid and 0.375% thiobarbituric acid. Butylated hydroxytoluene (0.01%) was added to the assay mixture to prevent autoxidation of the sample and the mixture was heated at 100°C for 15 minutes. After cooling, the mixture was centrifuged at 14,000 rpm for 5 minutes and the absorbance of the organic phase was measured at 535 nm. The amount of thiobarbituric acid reactive-substances (TBARS) was determined by the malondialdehyde standard curve and expressed as µmol/L in 100 µg protein.

### Measurement of SOD & GSH-Px Activities

SOD activity was detected according to previous report [48]. In this method, xanthine–xanthine oxidase complex produced superoxide radicals which reacted with water-soluble tetrazolium (WST-1). GSH-Px activity using H_2_O_2_ as a substrate was assayed by an adaptation of the method reported by Lawrence and Burk [49]–[51]. Using 96-well plate, SOD activity was measured at 450 nm and GSH-Px activity was measured at 412 nm by Microplate reader (Thermo Scientific, USA), and both of the data were presented as U/100 µg protein.

### Immunofluorescence

The specimens were cut into 14 µm sections. Standard immunofluorescence method for a double labeled immunofluorescence study was applied to identify the nerve and the neuropeptide as previous method [52]. Briefly, polyclonal anti-MAP2 antibody (Cell Signaling Technology, MA, USA) was used to stain the cutaneous nerves and polyclonal antibody for SP (Santa Cruz biotechnology, CA, USA) were used to stain the neuropeptides. The tissues were stained with anti-MAP2 antibody in a dilution of 1∶500 and with anti-SP antibody in a dilution of 1∶500. All sections were examined with Confocal Laser Scanning Microscope (Leica, Germany). Only structures with strong, continuous linear staining were counted as nerve fibers.

### Assessment of Hair Cycle

All mice were taken pictures with a digital camera (Panasonic, Japan) every day after depilation. The grayscale (0–255) of specific area in photograph (the region of neck) were analyzed by Image J software and presented as ratios (grayscale/255). The HE stain was used to quantify the stage of the hair follicles using a published classification technique based on the morphology of the dermal papilla and sebaceous glands [9]. To identify accurately, a minimum of 100 back skin hair follicles per mouse were scored which assigned to the telogen, anagen and catagen stages in ascending numerical order (anagen I–VI: 1–4; catagen I–VIII: 5–8; telogen: 9) according to their characteristic, stage-specific histological appearance as described in previous reports [10]–[11], [53]. The number of hair follicles in each specific hair cycle stage was then multiplied by its corresponding score. The resulting sum was divided by the total number of hair follicles per visual field to obtain the average hair cycle score which was defined in relevant hair cycle stage.

### Toluidine Blue Staining

Mast cells in the skin section were stained with toluidine blue. In brief, the deparaffinized sections were soaked in a solution containing 0.1 M citric acid, 0.2 M dibasic sodium phosphate and 0.5% toluidine blue for 20 minutes at room temperature. Mast cells were classified as degranulated when eight or more granules were found outside the cell membrane at 200×magnification as described previously [10], [16]. Number of positively stained cells per section of the entire dermis was counted and the ratio of degranulated mast cells to all mast cells was calculated.

### Statistical Analysis

All data were presented as the means ± S.E.M. Statistical significance between more than two groups was tested using two way ANOVA followed by the Newman-Keel test or an unpaired two tail Student's *t*-test. *P*<0.05 were considered statistically significant.

## Results

### CRS Induces Alteration of Hair Cycle

**Figure 1:**
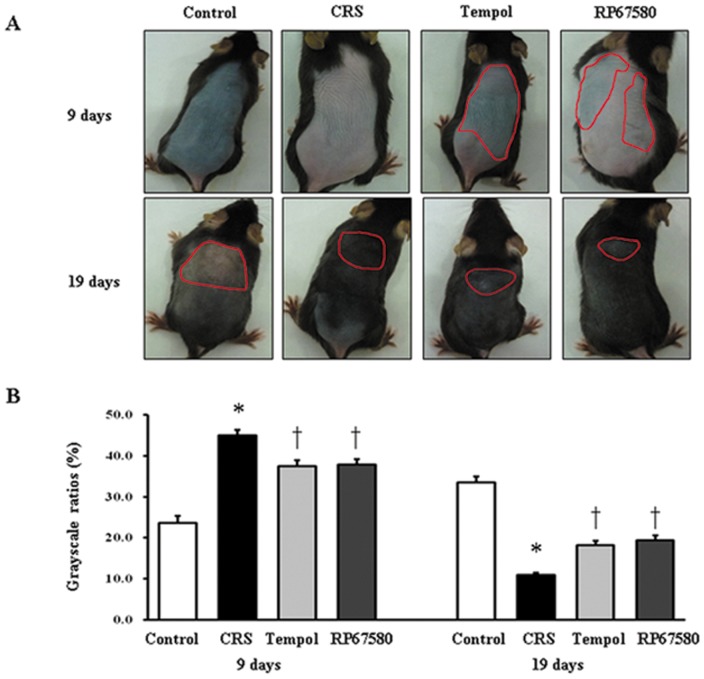
Macroscopic observations of the hair cycle. The hair shafts of murine skin were shaven by animal clippers beforehand. **A**: The significant area of color in the mouse dorsal skin was surrounded by a red line. **B**: The corresponding skin color gray-scale ratio on day 9 was shown on the left and day 19 on the right. Data were presented as mean ± SEM, n = 9 in each group, * *P*<0.05 compared with control group, † *P*<0.05 compare with CRS group.

**Figure 2:**
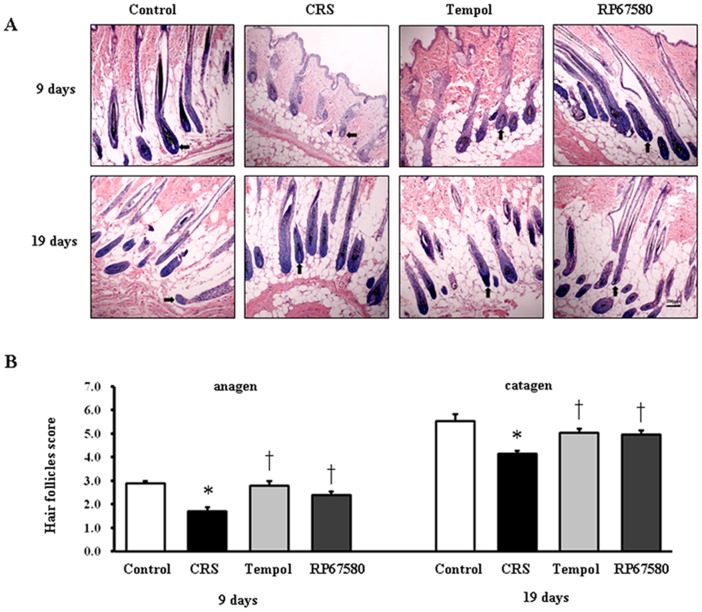
Effects of stress on the hair cycle stage. **A**: A representative area of each group on day 9 and day 19 after depilation with the majority of hair follicles. Original magnification was×100. **B**: The results of hair follicles score for day 9 were shown on the left and the data for day 19 on the right. The Y axis depicted histometric score assessed anagen induction. For each mouse a minimum of 10 individual visual fields were assigned to define hair cycle stages. Data were presented as mean ± SEM, n = 9 in each group, * *P*<0.05 compared with control group, † *P*<0.05 compared with CRS group.

To investigate whether CRS affects the hair growth to influence hair cycling in vivo, the onset and termination of anagen stage after depilation were examined in mice exposed to CRS compared to control mice. On day 9 after depilation (late anagen), the skin color of neck region in control mice was darker than the CRS mice (Figure 1A) and the corresponding skin grayscale ratio was significantly lower than CRS mice (Figure 1B). Morphological study showed most of hair follicles in control mice entered anagen V/VI and the majority of hair follicles in CRS mice was in anagen II/III (Figure 2A). On day 19 after depilation (catagen), skin color of neck region in control mice was lighter than the CRS group (Figure 1A) and the corresponding skin grayscale ratio was significantly higher than CRS mice (Figure 1B); meanwhile, most of hair follicles in control mice entered catagen V/VI, CRS mice was still in anagen VI (Figure 2A). On day 9 and day 19, scores of hair cycle in control mice were both significantly higher than in CRS mice (Figure 2B). These results suggested CRS inhibited hair growth by prolonging the telogen stage and delaying subsequent anagen and catagen stage.

### CRS Inhibits Mice Body Weight Gain and Increases Plasma Corticosterone Concentration

**Figure 3:**
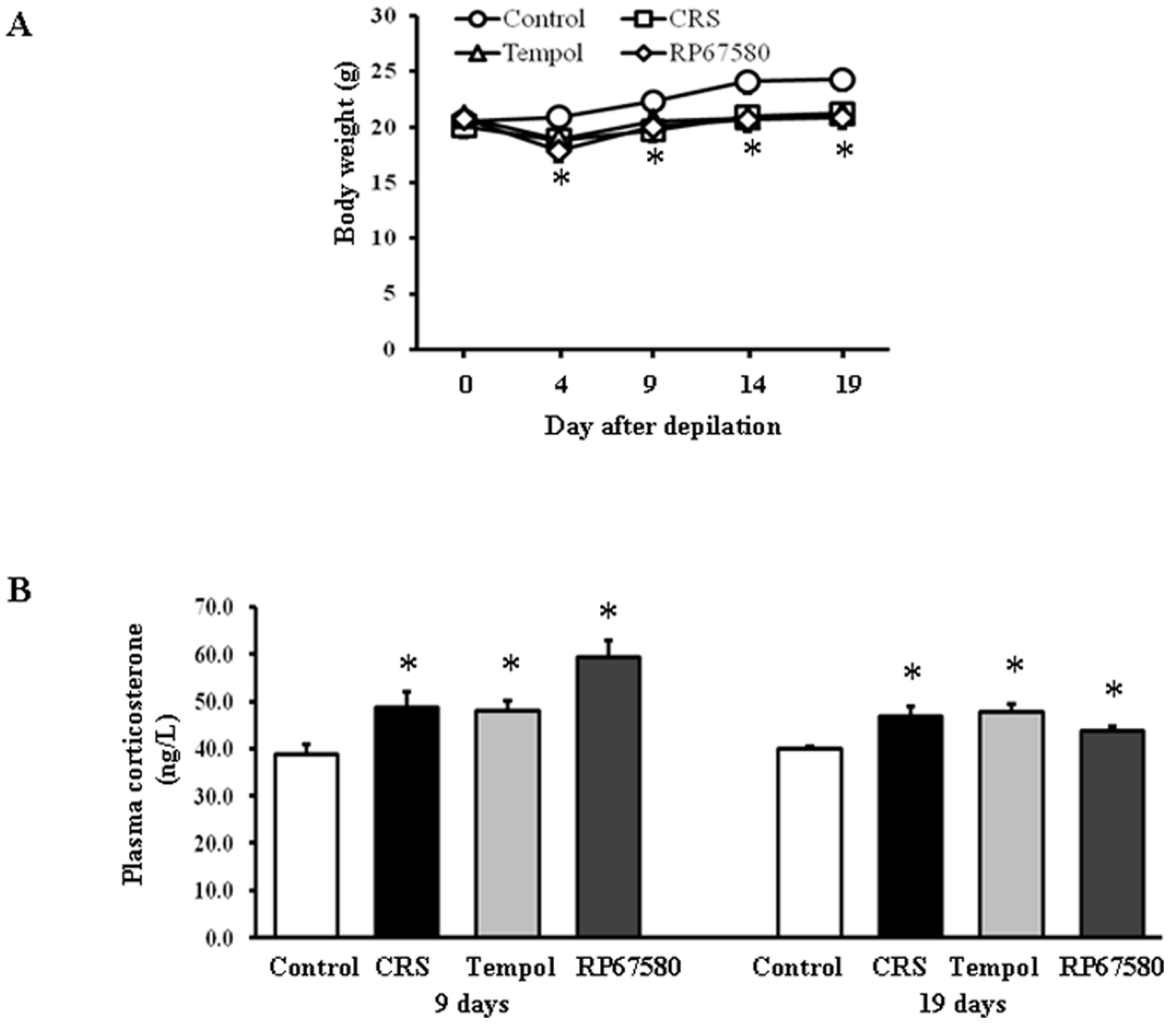
Effects of CRS on mice body weight gain and plasma corticosterone concentrations. **A**: On day 4, 9, 14, and 19 after depilation, body weight gain was significantly inhibited in CRS group compared with control group. **B**: The plasma corticosterone concentrations of mice for day 9 were shown on the left and the data for day 19 on the right. Data are presented as mean ± SEM, n = 9 in each group, * *P*<0.05 compared with control group.

To confirm whetherCRS successfully induced psychoemtional stress, we monitored mice body weight gain and measured plasma corticosterone concentration. Our results showed that CRS inhibited mice body weight gain significantly on day 4, 9, 14 and 19 compared with control group (Figure 3A). Plasma concentrations of corticosterone on day 9 and day 19 after depilation both significantly increased in CRS group compared with control group (Figure 3B). These results confirmed that CRS indeed induced psychoemotional stress.

### CRS Increases TBARS Levels and Decreases SOD&GSH-Px Activities in Skin

**Figure 4:**
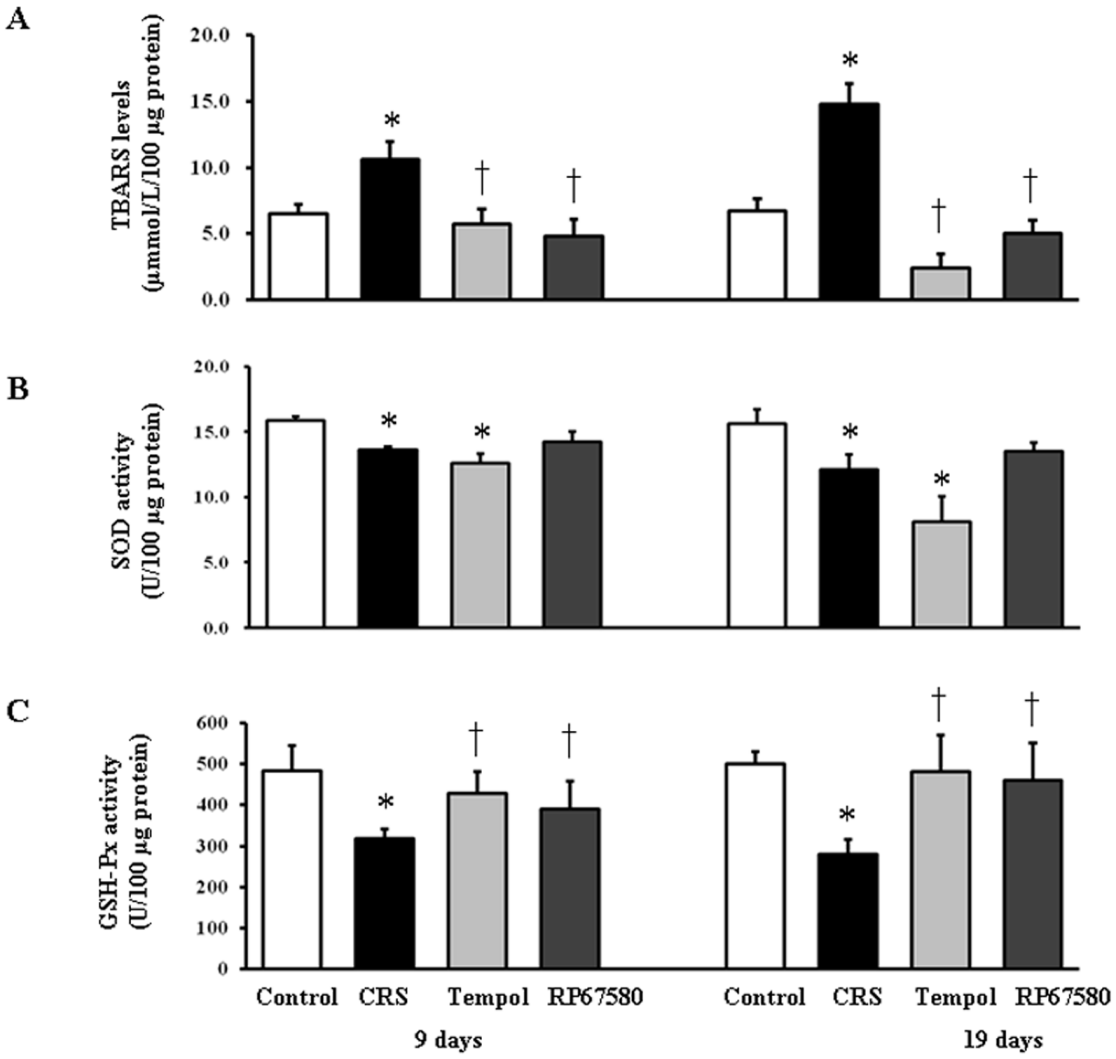
CRS induces skin tissue oxidative stress. **A**: TBARS levels in mice skin tissue on day 9 shown on the left and on day 19 shown on the right. **B**: SOD activities in skin on day 9 shown on the left and on day 19 shown on the right. **C**: GSH-Px activities in skin on day 9 shown on the left and on day 19 shown on the right. Data were presented as mean ± SEM, n = 9 in each group, * *P*<0.05 compared with control group, † *P*<0.05 compared with CRS group.

The effects of CRS on oxidative stress in skin tissue were examined by measurements of skin tissue lipid peroxidation marker, TBARS levels. On day 9 and day 19 after depilation, TBARS levels in CRS group were significant increased compared with control group (Figure 4A). Activities of SOD and GSH-Px in CRS group were markedly decreased than control group in skin (Figure 4B, C). These results indicated that CRS induced increase of oxidative stress might be through reduction of antioxidant enzyme activity.

### CRS Induces activation of SP-Mast Cell Pathway

**Figure 5:**
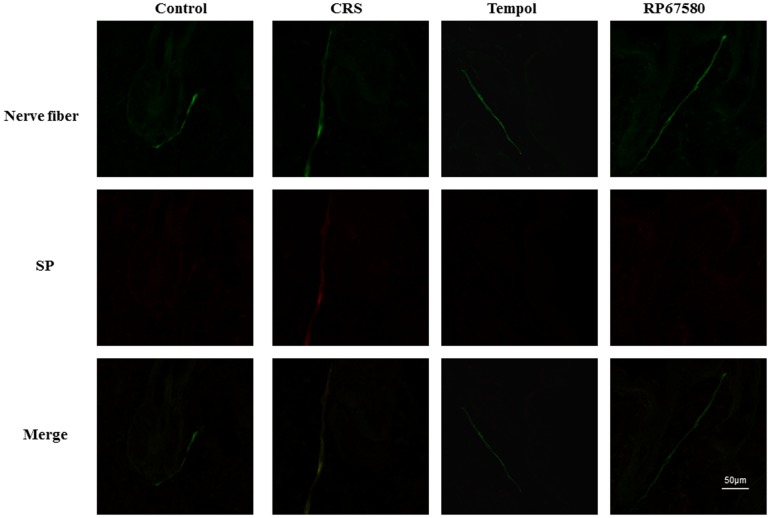
CRS increases amount of SP+ nerve fibers in dermis on day 9. A representative area of dermis in each group on day 9 after depilation was shown. The nerve fiber was stained continuous green linear and SP+ nerve fiber was stained red. Original magnification was×500. n = 6 in each group,

**Figure 6:**
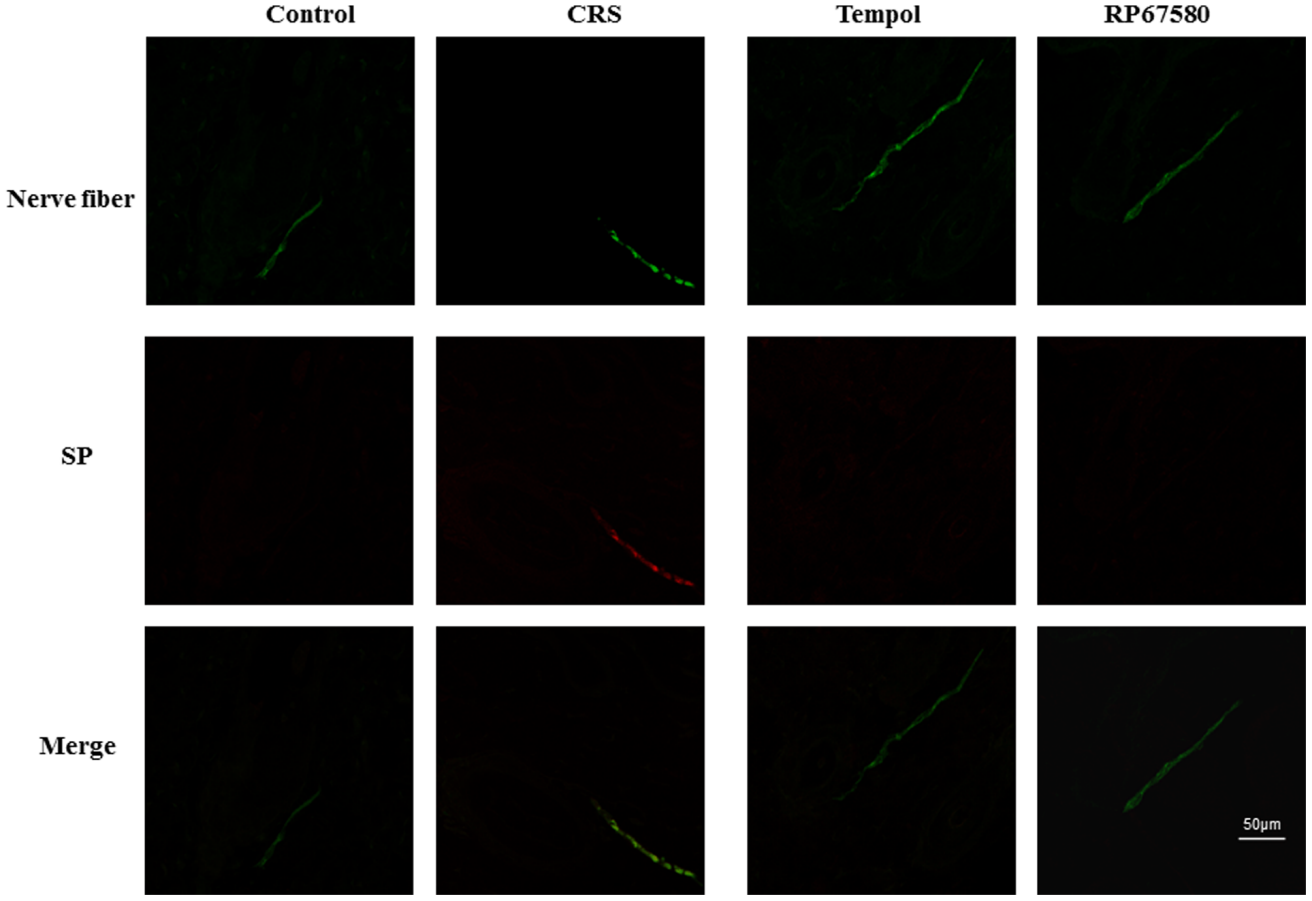
CRS increases amount of SP+ nerve fibers in dermis on day 19. A representative area of dermis in each group on day 19 after depilation was shown. The nerve fiber was stained continuous green linear and SP+ nerve fiber was stained red. Original magnification was×500. n = 6 in each group.

**Figure 7:**
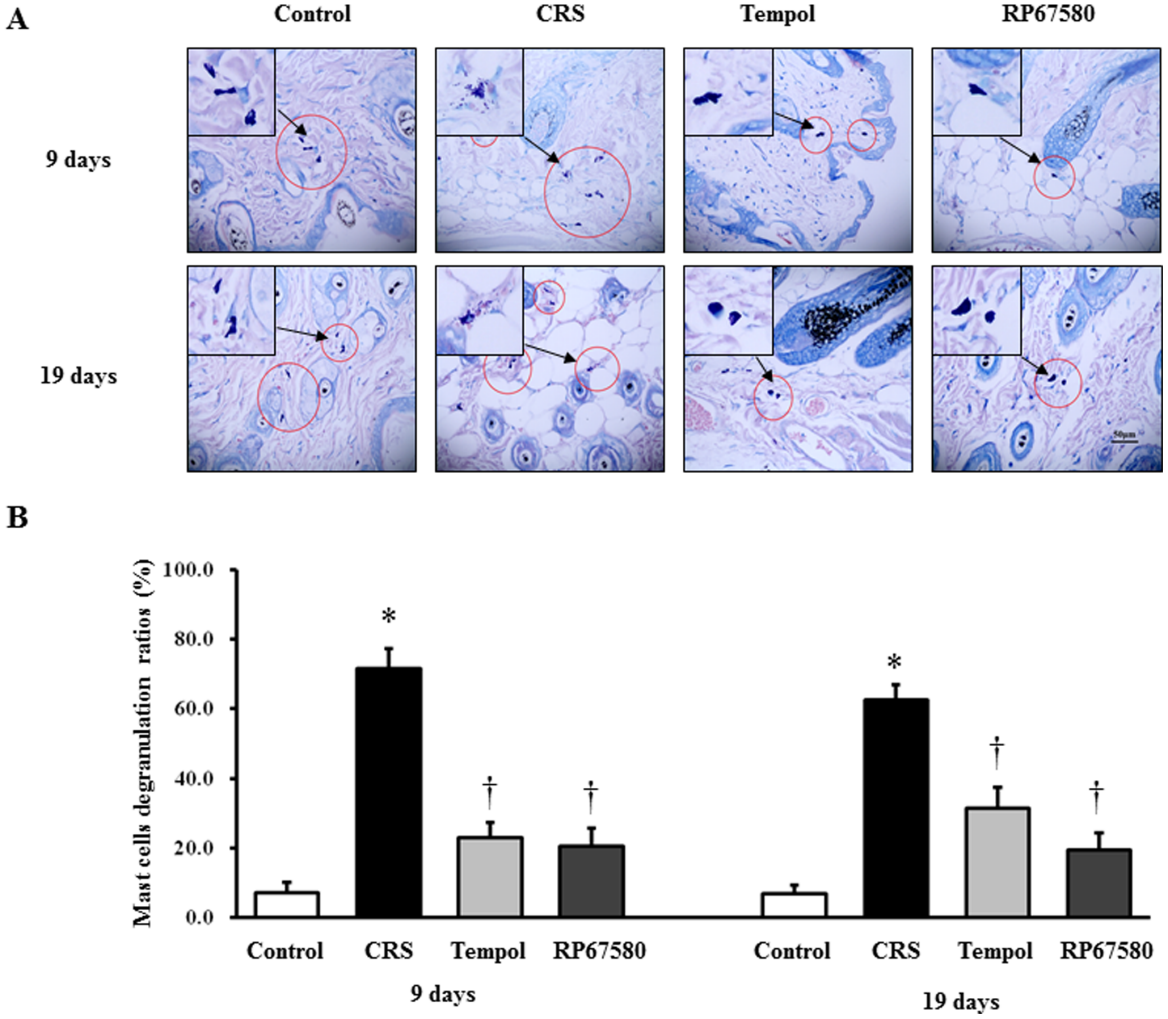
Effects of CRS on skin mast cell activation in dermis. **A**: A representative area of dermis in each group on day 9 and day 19 after depilation was shown. Original magnificaiton was×400. Dark blue stained cells are mast cells. **B**: The degranulated mast cells in each group on day 9 were shown on the left and day 19 on the right. Data were presented as mean ± SEM, n = 6 in each group, * *P*<0.05 compared with control group, † *P*<0.05 compared with CRS group.

To confirm the CRS induces increase of SP expression in peripheral nerve fibers, SP and nerve fibers marker, MAP2 were co-immunostained in the dermis. On day 9 and day 19 after depilation, the number of SP+ nerve fibers was increased significantly in CRS mice than in control mice (Figure 5, 6). Furthermore, to confirm CRS induces mast cell activation, we performed mast cell specific staining, toluidine blue staining. Results showed that the ratio of degranulated mast cells also increased significantly in CRS mice compared with control mice (Figure 7). These results suggested that CRS induced increase of peripheral nerve SP expression and inflammatory response which is concordant with previous reports [4]–[5].

### NK1 Receptor Antagonist Normalizes Most of CRS-Induced Alterations

To investigate the possible mechanisms involved in CRS-induced alterations in mice skin, NK1 receptor antagonist RP67580 was intraperitoneal injected in CRS mice. Although RP67580 did not influence CRS-induced decrease of body weight gain and increase of plasma concentrations of corticosterone (Figure 3A, 3B), but RP67580 significantly ameliorated CRS-induced alterations in skin gray scale and hair follicle score both on day 9 and day 19 (Figure 1 and Figure 2). In addition, administration of RP67580 significantly reduced CRS induced increase of SP+ nerve fibers number (Figure 5, 6), and inhibited increases of ratio in degranulated mast cells (Figure 7) in dermis both on day 9 and day 19.

Furthermore, RP67580 also normalized CRS-induced increase of TBARB levels and reduction of SOD and GSH-Px activities (Figure 4A, B, C). These results suggested that CSR-induced alterations were mediated by SP-NK1-mast cell-ROS pathway.

### Antioxidant Normalizes Most of CRS-Induced Alterations

**Figure 8:**
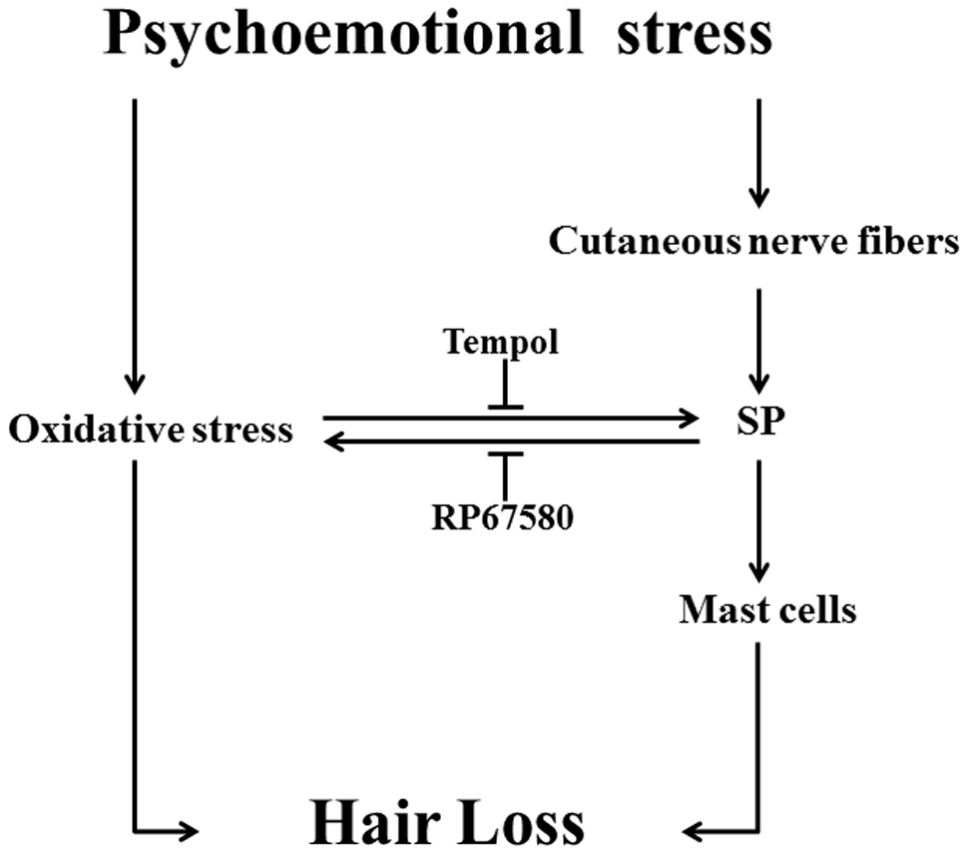
Schematic diagram of oxidative stress affects hair follicles cycling induced by psychological stress. Oxidative stress delay the anagen of hair cycle and activate SP-Mast cell pathway, and SP also affects oxidative stress in skin.

To investigate the possible role of ROS in CRS-induced SP-mast cell pathway activation, antioxidant Tempol was intraperitoneal injected in CRS mice. Our results showed that Tempol also did not affects CRS-induced decrease of body weight gain and increase of plasma concentration of corticosterone (Figure 3A, 3B), but significantly ameliorated CRS-induced alterations in skin gray scale and hair follicle score both on day 9 and day 19 (Figure 1 and Figure 2). As a strong antioxidant, Tempol reasonable reduces CRS-induced increase of TBARB levels. Our results also showed Tempol could increase CRS-induced reduction of GSH-Px activity but did not affect CRS-induced reduction of SOD activity (Figure 4A, B, C). Last but not the least, we found that Tempol significantly reduced CRS induced increases of SP+ nerve fibers number (Figure 5, 6) and ratio in degranulated mast cells (Figure 7) compared with CRS group on day 9 and day 19. These results clearly showed that ROS regulated CRS-induced alterations not only in the down-stream of SP-mast cell pathway, but also in the up-stream of SP-mast cell pathway (Figure 8).

## Discussion

One of the main findings of the previous studies was that psychoemotional stress could inhibit hair growth in vivo through SP-Mast cell pathway [4]–[5]. Using the mouse model of CRS, we firstly demonstrated the complicated role of ROS in regulation of this pathway by two aspects. One is through SP induces mast cell to generate ROS to exert pathological effects while the other is through other possible ligands, such as glucoticoid, cortisol to stimulate ROS generation, thereafter regulates SP synthesis.

Skin oxidative stress inhibited hair growth and delayed onset of the anagen in mice hair cycle that induced by CRS, including change of hair follicles morphology and skin color which were reversed by administration of antioxidant Tempol, confirming the undoubted role of oxidative stress in psychoemotional stress induced abnormal of hair growth (Figure 1 & Figrue 2). In order to investigate the whole changes of hair cycling, we chose two time points for observation. The first one is on day 9 after depilation while hair follicles of control mice enter into late anagen; The second one is on day 19 after depilation while hair follicles of control mice just enter catagen [9]–[10]. Compared withpreviously studies, we find that CRS delays both anagen and catagen in first hair cycle after depilation which has been considered refractory to systemic stress [11]. Furthermore, we do not observe the premature catagen in CRS model compared with sonic stress for 24 hours [4]. This discrepancy may be due to the different models used in the experiments. Our model started the stress exposure on day 1 after depilation (thus at onset of anagen development) and ended it on day 19 when catagen could be expected after anagen onset on day 1. This covers the entire time during which depilation induced hair cycle should take place. Previous studies started expouse of stress in mice when that was in anagen VI [4] or in the telogen phase [11] and did than not enter anagen. This phenomenon indicated that: (1) hair cycle is likely to be more sensitive to restraint stress than foot shock and CRS may be an appropriate model for hair cycling research. (2) Chronic and acute stimulation may have different effects on hair cycling. (3) CRS could not shorten but prolong the telogen stage and delay subsequent anagen and catagen stage.

Recently, the role of oxidative stress in skin disorders has been characterized. Many pathogenesis of skin disease might be attributed to ROS and oxidative stress, especially in hair aging and skin carcinogenesis [25], [54]–[55]. We showed here for the first time that chronic psychoemotional stress causes oxidative stress in mice dorsal skin by measurement of TBARS levels, SOD and GSH-Px activity. The results showed that on day 9 and day 19 after depilation, skin tissues of the stressed mice had high TBARS levels and low activity of SOD and GSH-Px compared with control mice. This indicated that CRS enhanced lipid peroxidation and production of ROS and inhibited the antioxidant system in skin. Moreover, the activity of SOD decreased significantly in mice treated with Tempol compared to control mice. This phenomenon may be due to that Tempol is a SOD mimetic and there may be a competitive inhibition between exogenous Tempol and endogenous SOD.

In the present study, we chose C57BL/6 mice for animal model because this strain is an ideal model for hair follicle research. Depilation induced anagen development and consecutive stages (catagen and telogen) were no significant difference with the natural hair cycling in C57BL/6 mice [56]. Moreover, the melanin of C57BL/6 mice only synthesized in the anagen lead to the skin color varies periodically. Therefore, we can roughly determine the hair cycling by mouse skin color [57].

Unlike the other models which have been described for studies of hair cycling constantly such as sonic stress for 24 hours [4]–[5] or foot shock for 2–4 weeks [11]–[12], we now used an animal model of chronic restraint stress (CRS) to research whether oxidative stress is implicated in progression of hair loss. CRS is an emotional stress which contains more psychoemotional component and can well simulated social psychological stressors by anxiety and fear in animals and can also increase plasma corticosterone concentration and inhibit body weight gain [33], [38]. Meanwhile, we applied antioxidant to inhibit the effects of oxidative stress to observe if antioxidants have any therapeutic effects in psychoemotional stress induced pathological changes, focused on changes of hair cycle.

Similar with previous studies [4]–[5], we also observed that CRS increased amount of SP+ nerve fibers, degranulation ratio of mast cells. Furthermore, we also observed CRS increased TBARS levels, decreased antioxidant enzyme activities. CRS induced alterations can be reversed by NK1 receptor antagonist. These findings suggest that SP could change local tissue redox condition by NK1 receptor which is widely expressed in mast cell, thereafter, it is reasonable to speculate that CRS induces SP-NK1-ROS pathway which may be similar with previous reported mechanisms of SP in nociceptive neurons [38]. In addition, we also observed that antioxidant could affect SP expression, suggesting ROS regulated CRS induced increase of SP expression. Although in the present study we did not demonstrate how CRS increases ROS thereafter mediate SP expression, according to well established stress-response theory, we reasonably speculate CRS increase stress-induced hormones through classic hypothalamic-pituitary-adrenal (HPA) axis and these hormones could stimulate ROS generation [58], thereafter to affect SP expression in peripheral nerves.

In addition to physical immobilization, psychoemotional stress plays a significant part in CRS [59]. After chronic restraint, whether on day 9 or day 19 after depilation compared with control mice, the stressed mice body weight gain was obviously inhibited and the concentration of corticosterone in the plasma was significantly increased, which suggest that mice are under stressful conditions [11]. CRS increased plasma corticosterone concentration have been examined in the greatest detail and have been seem as an indicator to measure systemic stress response [34], [59]–[60]. This indicated that psychoemotional factor, at least, plays a role in a series of changes of stressed mice, and the above evidence suggest that CRS model is successfully established in our research. Although administration of Tempol or RP 67580 could not reduce plasma corticosterone concentration, suggesting that antioxidant and SP receptor antagonist have few effects on systemic stress responses, at least we explore its possible therapeutic effects in CRS-induced hair loss. Limitation of the present study is CRS-induced loss of body weight gain may also affect hair growth.

Recent clinical trials have confirmed that the activities of GSH-Px and SOD were increased in scalp of patients with alopecia areata [61]. Relatively, the activity of GSH-Px in plasma of patients with alopecia was significantly decreased [62]. These clinical evidences indicated that antioxidant enzyme system played a critical role in the occurrence and development of alopecia. Our study now reports the first experimental evidence that oxidative stress involved in the abnormal of hair cycle as well as its role in regulation of SP-Mast cell pathway. The present data provide a convincing rationale for antioxidant application in clinical treatment with psychoemotional stress induced hair growth disorders.
